# Rolling out Rwanda’s national palliative care programme

**DOI:** 10.2471/BLT.18.031118

**Published:** 2018-11-01

## Abstract

Only 14% of people worldwide who need palliative care receive it. Christian Ntizimira talks to Tatum Anderson about the challenges of applying Western models of palliative care in an African setting.

Q: How did you become interested in palliative care?

A: After graduating, my dream was to be a surgeon, because I saw so many people who had suffered after the genocide against the Tutsi in 1994.That dream changed after I worked in internal medicine at Kibagabaga Hospital and met a 24-year-old patient who was dying of liver cancer. He was in a separate room because he was screaming with pain. The patient’s mother knelt before me and begged me to do something. In Africa, it is not customary for an older person to kneel before a young person. I felt helpless and frustrated. I had never prescribed morphine before and anyway it wasn’t readily available for cancer patients in the hospital. The protocol for prescribing morphine was highly restrictive: a prescription had to be written out in red ink and signed by three or four people. That night the young man died in a horrible state and his family was inconsolable. This made a deep impression on me. “Why become a physician if my role is just to watch patients suffer?” I asked myself.

Q: What training was available in palliative care at the time?

A: A year later, in 2009, I participated in 10 days training in palliative care, organized by two international nongovernmental organisations, IntraHealth and Mildmay, in collaboration with the Rwandan health ministry. The focus was on palliative care for patients with the end-of-life diseases associated with HIV. After that training I knew palliative care was my calling.

Q: How did you introduce palliative care into your hospital?

A: When I became the director of Kibagabaga Hospital, I made several changes to improve the quality of life of patients with life-threatening illnesses. One was to make palliative care, particularly treatment with morphine, accessible for end-of-life patients with severe pain. The idea was to integrate palliative care into other programmes and not to create a separate palliative care department. We started training nurses, physicians, physiotherapists and psychologists to work as a team to provide patients with palliative care. The physicians learned to treat patients’ side-effects, such as constipation and anxiety, when prescribing morphine. Team members were also trained in the provision of psychosocial support to both the patient and family. In addition, administrative staff, ambulance drivers and cleaners were trained, for example, to better support patients when they arrived at the hospital.

“After that training I knew palliative care was my calling.”

Q: What were the challenges?

A: The perception and attitudes of many health-care providers, as they were not immediately convinced of the benefits of accompanying and treating dying patients, was a challenge. Physicians sat at the same table with nurses, social workers, anaesthetists and physiotherapists – for the first time – to discuss and decide together how their patients should be managed. Some physicians found it difficult to accept this new way of working. However, after the physicians completed the palliative care training provided by the health ministry, they were much less resistant.

Q: How were you able to increase access to morphine for patients in pain?

A: We trained physicians to prescribe morphine and changed the protocol, thus reducing the need for the red pen and the many signatures for just one ampoule of morphine. We also increased morphine supplies in hospitals, as before – anaesthetists for post –operative pain only used morphine. We had to request more morphine from the health ministry for moderate and severe pain of cancer patients and other patients in end-of-life care. Pain management is the backbone of palliative care, so all physicians in our hospitals were trained to prescribe morphine, not just the palliative specialists. We encouraged all departments to use the World Health Organization (WHO) guidelines, based on the WHO pain ladder, to ensure that health providers recognized pain as a symptom, just like a fever. In addition, nurses trained in palliative care were also allowed to prescribe some treatments.

Q: How did you become interested in community palliative care?

A: In the past, most patients preferred to die in the hospital, saying: “If I go home who will take care of me?” We did a survey of palliative care patients at Kibagabaga Hospital. More than 70% of them said they would rather go home if they could receive palliative services at home. So, the challenge was how to provide these services at the community level. One health ministry-approved project, implemented in 2014, attempted to address this question by training two nurses at each health centre in our hospital catchment area and one community health worker per village (481 in total). The idea was to provide patients with the appropriate support at each level of the public health system after their discharge from hospital.

Q: How did you bring community health workers into palliative care?

A: We asked the person in charge of community health workers at the hospital to help us to identify one community health worker in each village to identify people in need of palliative care, thus supporting a triage system at the community level aimed to reduce the flow of patients at the district level. After two months, these workers identified almost 150 patients who lived near Kibagabaga Hospital with cancer, HIV, renal failure, heart failure and progressive neurologic disease.

Q: How do you manage pain relief at the community level?

A: As mentioned, more than 70% of our end-of-life patients opt to go home. When we discharge them, we know who they are, where they live and who takes care of them. We also know the morphine dose the patients need and when they will need more. In the past, we could only prescribe injectable morphine at Kibagabaga Hospital. Now we are using morphine tablets which patients can take at home, and their caregivers can renew the prescription for pain relief for least for a month at the hospital. If the patients have nausea or vomiting, they can go to the health centre for relief. Before, it was a nightmare for the patient: firstly, there was no morphine available and secondly, they had to manage to get paracetamol or ibuprofen prescribed at the health centres or private pharmacies. Now, Rwanda produces its own morphine syrup and every district pharmacy can request the quantities of morphine needed in the district from the Rwanda Biomedical Center, and it is free for patients.

Q: What were the challenges for increasing access to morphine?

A: The health ministry asked us why we were requesting more morphine supplies. Naturally they were concerned that it might be diverted illegally. I explained that we had many cancer patients with moderate and severe pain who needed palliative care services. Another problem, at first, was obtaining morphine tablets, because only injectable formulations were available at the hospital.

“When you are well, you belong to yourself. When you are sick, you belong to your family.”

Q: What progress have you made rolling out palliative care across the country?

A: In 2017, the health ministry established a new cadre of health professionals as part of the home-based care practitioners’ programmes. They provide a range of services including palliative care. The law governing narcotics drugs, psychotropic substances and precursors in Rwanda changed in 2012 to allow nurses and other health providers trained in opioid prescription to prescribe morphine. In Rwanda, we have made so much progress in the provision of palliative care, but there are still challenges in service delivery.

Q: What was the outcome of the project at Kibagabaga?

A: Kibagabaga Hospital started Rwanda’s first paediatric palliative care programme in 2009. Then in 2011, Rwanda became one of the first countries in Africa to launch a national policy for palliative care: a four-year plan that included an implementation programme. Our work at Kibagabaga served as an inspiration for hospitals across the country. The national plan stated that all Rwandans in need of end-of-life care should have access to high quality, affordable palliative care services to meet their physical, psychological, social and spiritual needs by 2020. Then, in 2012, the government launched a national training programme in palliative care. Since then, our multidisciplinary team at Kibagabaga has trained people from the district and teaching hospitals across the country.

Q: What have you learnt, about delivering palliative care in Rwanda?

A: When I studied palliative care, I realised that many aspects of Western palliative care may not be appropriate in the Rwandan context and that palliative care needs to be adapted and aligned with our cultural values. For instance, in the USA, the “patient’s autonomy” is very strong which means they can decide whether and to whom to disclose the details of their disease and treatment. In Rwanda, family caregivers often say: “When you are well, you belong to yourself. When you are sick, you belong to your family.” So, the patient’s autonomy is interconnected with the family’s decision and the community. In the USA, people talk about death more easily and patients are willing to sign a do-not resuscitate form. In Rwanda we talk about life until the end and it is not appropriate to talk about death when the patient is still breathing. A dying patient for us is a living patient. So I have had to weigh up which elements of palliative care we could apply, and what was legally or morally acceptable in Rwanda and what was not. I strongly believe that the concept of palliative care will contribute to restoring the sense of humanity and dignity lost during the genocide against Tutsi, because it is focused on the person and not only on the disease.

Q: What are you doing to avoid opioid dependency and overdoses in Rwanda?

A: Drugs like naloxone are available to reverse the effects of an overdose, but overdoses are extremely rare, because we start with very low doses. Dependency is not an issue in Rwanda, because we are treating patients at a very late stage of their illness..

